# Innovation in Isolation? COVID-19 Lockdown Stringency and Culture-Innovation Relationships

**DOI:** 10.3389/fpsyg.2021.593359

**Published:** 2021-02-03

**Authors:** Hansika Kapoor, Arunima Ticku, Anirudh Tagat, Sampada Karandikar

**Affiliations:** ^1^Monk Prayogshala, Mumbai, India; ^2^Neag School of Education, University of Connecticut, Mansfield, CT, United States

**Keywords:** COVID-19, creativity, cultural dimensions, innovation, pandemic, stringency index

## Abstract

In a bid to curb the spread of COVID-19 in 2020, several countries implemented lockdown procedures to varying degrees. This article sought to examine the extent to which country-level strictness, as measured by the Government Response Stringency Index (2020), moderated the relationship between certain cultural dimensions and estimates of national innovation. Data on 84 countries were collated for Hofstede’s cultural dimensions (2015), and from the Global Innovation Index (2020). Owing to the robust relationships between innovation and the dimensions of uncertainty avoidance, power distance, and individualism, these were used in moderation analyses. In general, power distance was inversely related to innovation, whereas individualism was directly related to it. Results indicated that collectivist and high power distance countries showed lower innovation, irrespective of levels of government stringency as a response to COVID-19. On the other hand, among individualistic and low power distance countries, lower innovation was associated with increased stringency (e.g., blanket restrictions on movement). Higher innovation was observed when such countries had a less severe government response. The dimension of uncertainty avoidance was not significantly associated with innovation at the country level. The implications of lockdowns on general innovation, its inputs, and outputs are discussed in the context of cultural dimensions and country-level policies.

## Introduction

On March 11, 2020, the World Health Organization characterized COVID-19 as a global pandemic. At the time of this writing (December 2020), over 75 million cases have been reported and over 1.5 million deaths have been recorded (Dong et al., 2020). Combating the spread of the virus entails enforcing strict social distancing measures, with most national and local governments implementing lockdowns and stay-at-home orders.

In this context, the Oxford COVID-19 government response tracker (Hale et al., 2020) identified nine distinct factors pertinent to lockdowns, which assess the overall stringency of the measures. These include closing of schools, closing of workplaces, cancelation of public events, restrictions on gatherings, closing down public transport, restricting movement with notifications to stay at home, and restrictions on overall travel. While citizens are permitted access to essential services (such as grocery and pharmacy stores) in most countries, there are typically strict requirements such as maintaining social distancing and restrictions on the number of people who can visit a store at a time. Higher stringency is directly associated with reducing the overall mobility of individuals across the board. For instance, Hussain (2020) found that a country’s successful performance on social-distancing has been associated with higher stringency levels therein. Strict interventions were also associated with bringing about behavioral changes within citizens to implement social distancing in various countries (Morita et al., 2020). Simulated models and observations of limited country-level data allude to a reduction in overall cases and better control of the pandemic, if strict measures are implemented and adhered to (e.g., Gatto et al., 2020; Tian et al., 2020).

Stringency of government measures has also been found to be associated with a reduction in stock market returns (Ashraf, 2020), as well as a slowdown in other measures of economic activity such as energy consumption, mobility, and industrial production (Deb et al., 2020). Furthermore, as policy stringency associated with COVID-19 varied by the national capacity to implement it, it is likely to have had diverse effects on outcomes across countries (Capano et al., 2020). Early estimates suggested a nearly 10 percent reduction in economic activity across Europe and Central Asia (Demirguc-Kunt et al., 2020). Thus, government measures to contain mobility, ensure social distancing, and mask-wearing (non-pharmaceutical interventions) are likely to have constrained the opportunities for innovation in the short run.

### Confinement and Innovation

Overall stringency of lockdown measures directly or indirectly leads to home-based confinement, with social isolation being a predominant consequence. Being confined in this manner could cause boredom and have repercussions on one’s mental health, including stress, anxiety, depression, and PTSD (Huang and Zhao, 2020; Shevlin et al., 2020; Tang et al., 2020; Torales et al., 2020). Even when a household is not directly impacted by COVID-19, there may still be stressors associated with perceptions about the current situation, or economic and other concerns that have resulted from the pandemic. Such worries may be exacerbated by imposition of self-isolation (Sabat et al., 2020). Higher uncertainty may also be detrimental to mental health, causing excessive stress, frustration, and anxiety (Schwartz, 2004).

Engaging in creative processes^1^ and activities is one way of relieving such negative affect. The rise in everyday creativity during this time is evident through general social media content, where more and more individuals are showcasing their at-home talents, including novel ways to experiment with daily tasks like taking out the garbage (Kapoor and Kaufman, 2020). Research on creativity within a sample of undergraduate students found that engaging in dance/art therapy was associated with having more positive coping mechanisms (Logan, 2016). Whereas everyday creativity has been found to predict positive emotions within an adult population (Conner et al., 2018), creativity in the form of indulgence in poetry-writing is also a helpful coping strategy in lockdown (Carroll, 2005). Moreover, creativity has been found to be a potential protective factor for overall household resilience, particularly in times of prolonged isolation (e.g., Verger et al., 2020). Given its affective benefits, it is imperative that individuals are able to engage in creative activities within a constrained environment. Moreover, feelings of boredom within pandemic-related isolation can potentially deter adherence to safety-measures (Martarelli and Wolff, 2020; Wolff et al., 2020), which is why creative engagement could directly buffer against the effects of the pandemic.

Data also suggest that national creativity and innovation parameters are interlinked with overall subjective well-being at the country level (Kapoor and Tagat, 2017). Studies comparing country-level innovation and creativity are often conflicting, with some contending that Eastern countries are likely to score lower on overall creativity as compared to western nations (Xie and Paik, 2018). This is often attributed to the cultural make-up of these nations and the classic individualism versus collectivism argument (Hofstede’s dimensions; Hofstede, 1984). Complementing evidence suggests that Western countries value novelty, whereas Eastern countries value usefulness (Morris and Leung, 2010). This was also observed empirically wherein American and Taiwanese teams were found to be equally original when generating ideas. However, Taiwanese teams tended to discard novel ideas, whereas American teams were likely to further develop such thinking (Liou and Nisbett, 2011). These parameters suggest that perceptions toward innovative and creative output vary cross-culturally. Therefore, understanding innovation requires looking at the bigger picture, within the context of culture.

### Cultural Dimensions and Innovation

According to Hofstede (2011), culture is a “collective programing of the mind that distinguishes the members of one group or category of people from others” (p. 3). This indicates that culture is a group-determined, collective phenomenon with certain defining characteristics. National culture has been theorized to differ along power distance, uncertainty avoidance, individualism versus collectivism, masculinity versus femininity, long term orientation, and indulgence versus restraint (Hofstede et al., 2010). Zha et al. (2006) contend that cultures differ in the extent to which they value and nurture creative potential in their citizenry. They concluded that culture influences such potential in four ways: how it is defined, the process or means by which it occurs, the domains it is likely to influence, and the degree to which it is nurtured (see also Sternberg and Lubart, 1996). This has been found to be especially true for power distance, uncertainty avoidance, and individualism versus collectivism (Shane, 1993).

Power distance refers to the extent to which less powerful members of social units (such as organizations and families) accept and expect that power is distributed unequally (Hofstede, 2011). East European, Latin, Asian, and African countries, being more power distant, value obedience to a greater degree as opposed to German, English-speaking Western countries that do not have a strict policy on the use of power.

Uncertainty avoidance or ambiguity tolerance indicates the extent to which a culture programs its members to feel either uncomfortable or comfortable in unstructured situations (Hofstede, 2011). Herein, cultures differ in the extent to which unstructured situations are perceived to be an inherent, continuous threat in life, and differ in the use of strict behavioral codes, laws and rules, and disapproval of deviant opinions to minimize such uncertain situations.

The dimension of individualism versus collectivism refers to “the degree to which people in a society are integrated into groups” (Hofstede, 2011, p. 11). Herein, cultures differ in the extent to which they emphasize group values and personal (for example, Western countries are individualistic) or group benefits (for example, Eastern countries are collectivistic).

Past literature has consistently noted the vital role played by culture in determining creativity and innovation (Westwood and Low, 2003; Shah, 2013; Hermida et al., 2019). Khan and Cox (2017) suggested that societies displaying higher individualism and indulgence, and lower masculinity were associated with more innovation (as measured by the Global Innovation Index; GII). Along with individualism (which has been linked with the size of the creative industry, Rinne et al., 2013), femininity, long-term orientation, indulgence, and low uncertainty avoidance were found to be key in aiding innovation (Prim et al., 2017). Further, lower uncertainty avoidance has been deemed necessary for innovation because innovation required tolerance for risk and change (Shane, 1993; Efrat, 2014). Higher individualism and lower power distance also contributed to innovation; the former due to its association with greater autonomy, independence, and freedom that facilitates creative thought (Shane, 1993), and the latter owing to the rejection of established social order and distribution of power (Shane, 1993; Varsakelis, 2001; Efrat, 2014; Xie and Paik, 2018).

Similarly, people primed for individualism (by asking questions about themselves and why it was advantageous to “stand out” of a group) showed greater group creativity than those primed for collectivism (by asking them questions about the social groups they belonged to and why it was advantageous to “blend in” in those groups; Goncalo and Staw, 2006). However, creativity present at the individual psychological level may not necessarily manifest at the country level within collectivist nations due to the lack of opportunity for people to freely express themselves. Moreover, there may be several barriers to creative expression if individuals are overly cautious about sharing their ideas, fearing rejection by those in power within a hierarchical structure.

Sarooghi et al. (2015) conducted a meta-analysis of 52 empirical studies with an aim to examine the link between creativity and innovation. Using regression analyses, they found that creative ideas are more often turned into innovative outputs at an individual level. Several studies indicate that collectivistic cultures are more likely to be successful in achieving innovation from creative ideas (see also Jones and Davis, 2000). Results also revealed that a moderate level of uncertainty avoidance is key to converting creativity to innovation, suggesting that moderate risk-taking and ability to overcome resistance may be instrumental in maximizing this relationship. Therefore, moderate levels of risk-taking and an ability to overcome resistance to change are inferred to be critical to not only idea generation, but also implementation (Sarooghi et al., 2015).

### The Present Study

Governments all over the world have adopted different strategies to contain the spread of COVID-19. At the very least, most of these measures have led to restricted mobility of people, spawning a sharp and sudden reduction in human activity, particularly productive economic activities (Bonaccorsi et al., 2020). However, such restrictions and stringency can have unintended consequences on innovative and creative behaviors, particularly at the national level. For example, in Germany, lack of clarity on government intervention stifled innovation from startups (Kuckertz et al., 2020). With respect to the current situation, a novel and life-threatening one, innovation is required in many facets including the provision of timely public health care responses (Cohen and Cromwell, 2020; Guest et al., 2020). Therefore, it is vital that creativity and subsequent innovation do not dampen during the pandemic as they are powerful strategies to buffer against decline in well-being. Additionally, government responses will need to consist of novel legal tools to ensure breadth as well as strength in dealing with COVID-19 (Parmet and Sinha, 2020). This indicates the need for creativity and innovation not only from national citizenry, but also policy actors and governments (Azoulay and Jones, 2020). However, stringency is likely to impact original output at such a time, perhaps more so for cultures that do not encourage creativity or innovation to a great extent.

Although past research has examined how cultural dimensions relate to individual and national creativity and innovation, findings were inconsistent (Harzing and Hofstede, 1996; Westwood and Low, 2003; Rinne et al., 2013; Sarooghi et al., 2015). Furthermore, the relationship between government policies and innovation remains ambiguous, especially in the context of a global pandemic. Layos and Peña (2020) argued that countries with well-established and efficient innovation systems may be expected to respond to a global health crisis better than others, owing to greater institutional preparedness, scientific knowledge, and favorable economic conditions.

Therefore, we propose a model that may help examine the interplay of cultural dimensions with stringency of policy, which may in turn influence the overall impact on national creativity and innovation. The primary aim of this investigation was to determine the associations between specific cultural dimensions and country-level indices of innovation.^2^ The values of low power distance, low uncertainty avoidance, and high individualism propagate the expression and sampling of novel ideas. These values foster exploration of counter-normative and alternative ideas, reduce the pressure to conform, and reduce ambiguity-induced anxiety (Kwan et al., 2018). Further, we proposed that national-level stringency as a response to the COVID-19 pandemic would moderate these relationships, based on the aforementioned literature.

**FIGURE 1:**
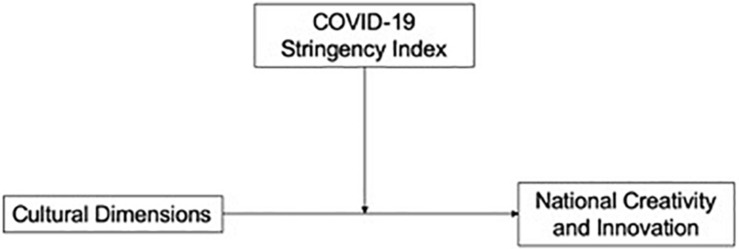
Theoretical model depicting the relationship between cultural dimensions and national creativity/innovation, moderated by the stringency index.

The overall model is presented in Figure 1. Here, the relationship between cultural dimensions and national creativity/innovation is determined on the basis of past literature. For instance, it is assumed that uncertainty avoidance and power distance are inversely related to innovation, whereas individualism is positively related to innovation (Shane, 1993; Efrat, 2014). As formulation of the stringency index has emerged recently owing to the varied state responses to the COVID-19 pandemic, we propose that the level of stringency imposed within a country will influence the magnitude of the relationship between specific cultural dimensions and national innovation. The index may not explain why this relationship exists, and will therefore not constitute a mediation. Yet, the index may explain how much the relationship changes as a result of the level of constraints on mobility, business, and travel. Moreover, different nations have had varying responses to the pandemic in terms of their confinement strategies. This variation is likely to capture cultural, sociopolitical, and economic disparities as well. In the context of the current model, creative industries, which are primary contributors to the creative economy and thereby innovative output, are likely to have suffered setbacks as well (Comunian and England, 2020).^3^

Thus, the following hypotheses were proposed:

H1: The stringency index moderates the inverse relationship between uncertainty avoidance and scores on the GII index, as well as GII inputs and outputs.
H2: The stringency index moderates the inverse relationship between power distance and scores on the GII index, as well as GII inputs and outputs.
H3: The stringency index moderates the direct relationship between individualism and scores on the GII index, as well as GII inputs and outputs.

## Materials and Methods

### Data Sources

Data on cultural dimensions (2015) comes from an earlier time period as compared to the data on innovation and stringency (2020). Thus, this study examines the moderating effect of a current moderator (stringency) on hypothesized relationships between cultural dimensions and global innovation; all data is collated at the country-level.

#### GII

Data on innovation were extracted from the Global Innovation Index (Dutta et al., 2020), which presents current trends in innovative behavior across 131 economies. The GII emphasizes the execution, implementation, and valuation of creative ideas, taking values between 0 and 100, with scores closer to 100 indicating higher country-level innovation.^4^ The GII also provided sub-indices of inputs and outputs of innovation. The former includes five pillars: institutions, human capital and research, infrastructure, market sophistication, and business sophistication; the latter includes knowledge and technology outputs and creative outputs. The parameters within the sub-indices provide a direct, official measure of innovation that captures both the environment within which innovation takes place as well as the result of innovative activities (Dutta et al., 2020). The average of the sub-indices comprises the overall GII Index. The GII 2020 dataset represents the most recently available data on this index.

#### Stringency Index

Data on government measures of stringency were taken from the Oxford COVID-19 Government Response Tracker’s Stringency Index (OxCGRT; Hale et al., 2020). The index consists of nine components, each measuring a different aspect of government stringency in response to the coronavirus. This paper uses the average value of the stringency index between January 1, 2020 (the earliest date that the index is available) and September 1, 2020 for each country to remain consistent with the GII data. It can take values between 0 and 100, with 100 representing the highest level of stringency imposed by a government.

#### Cultural Dimensions

Data on cultural dimensions were obtained from Hofstede’s Cultural Insights for 2015 (Hofstede Insights, 2020). The six dimensions on which data were available included (a) power distance; (b) individualism; (c) masculinity; (d) uncertainty avoidance; (e) long-term orientation; and (f) indulgence. Scores along these indices took values between 0 and 100, with a higher score indicating a stronger cultural parameter for each country. This data was then merged with the metrics from the GII and OxCGRT to generate a dataset for analysis.

## Results

Table 1 displays descriptive statistics for the measures used in the present study. Table 2 presents zero-order correlations between innovation, cultural dimensions, and the stringency index. The GII index, its inputs, and outputs were strongly and positively correlated. All innovation measures were inversely related to power distance, but directly to individualism. Further, higher the stringency in a country, lower was the innovation. To standardize the variables, *z* scores were computed and used in subsequent moderation analyses.

**TABLE 1:** Descriptive statistics for innovation indices, cultural dimensions, and the stringency index.

Variables	*N*	*M*	*SD*	Min	Max
GII	141	34.02	12.59	13.56	66.08
GII – Inputs	141	43.42	12.47	19.85	70.20
GII – Outputs	141	24.62	13.29	6.470	62.75
Uncertainty avoidance	108	64.39	21.27	8.00	100.00
Power distance	108	65.09	20.68	11.00	100.00
Individualism	108	38.33	21.54	6.00	91.00
Stringency index	171	52.79	12.36	10.69	74.80

*GII, Global innovation index.*

**TABLE 2:** Zero-order correlations between study variables.

	*1*	*2*	*3*	*4*	*5*	*6*	*7*
1. GII	1						
2. GII – Inputs	0.98***	1					
3. GII – Outputs	0.98***	0.91***	1				
4. Uncertainty avoidance	–0.11	–0.10	–0.12	1			
5. Power distance	−0.57***	−0.56***	−0.55***	0.18	1		
6. Individualism	0.70***	0.68***	0.69***	–0.12	−0.64***	1	
7. Stringency index	−0.21*	−0.19*	−0.22*	0.04	0.40***	−0.33***	1

*p < 0.05; ***p < 0.001.

*GII, Global Innovation Index.*

Data were analyzed using the Stata 16.1 and reproduced using the PROCESS macro on SPSS 23 (Hayes, 2017). Table 3 presents the moderation results, indicating significant interaction effects between stringency and cultural dimensions when predicting innovation indices. Contrary to expectations, uncertainty avoidance was not associated with innovation at the country-level (H1). The analysis indicated that 41% of the variance in GII was attributable to the main and interaction effects of power distance and stringency, *F*(3, 80) = 18.70, *p* < 0.001, *R*^2^ = 0.41; about 40% of the variance in GII inputs was explained by the same main and interaction effects, *F*(3, 80) = 18, *p* < 0.001, *R*^2^ = 0.40; and 39% of the variance in GII outputs was accounted for by power distance and stringency, *F*(3, 80) = 17.05, *p* < 0.001, *R*^2^ = 0.39 (H2). When individualism and stringency predicted the indices, 60% of the variance in GII, *F*(3, 80) = 39.65, *p* < 0.001, *R*^2^ = 0.60, 58% of the variance in GII inputs, *F*(3, 80) = 36.81, *p* < 0.001, *R*^2^ = 0.58, and 57% of the variance in GII outputs, *F*(3, 80) = 35.30, *p* < 0.001, *R*^2^ = 0.57, was attributable to the main and interaction effects (H3).

**TABLE 3:** Regression analysis predicting innovation indices from cultural dimensions, as moderated by the stringency index.

	GII		GII – Inputs		GII – Outputs	
			
Variables	*B*	*SE*	*B*	*SE*	*B*	*SE*
Uncertainty avoidance	–0.09	0.11	–0.07	0.11	–0.09	0.11
Stringency index	−0.39**	0.12	−0.37**	0.12	−0.40**	0.12
Uncertainty avoidance × stringency	–0.09	0.13	–0.07	0.13	–0.12	0.13
Power distance	−0.55***	0.09	−0.54***	0.09	−0.53***	0.09
Stringency index	0.16	0.11	0.14	0.11	–0.17	0.11
Power distance × stringency	0.25*	0.12	0.23	0.12	0.25*	0.12
Individualism	0.69***	0.07	0.67***	0.07	0.67***	0.07
Stringency index	–0.16	0.09	–0.14	0.09	–0.17	0.09
Individualism × stringency	−0.26*	0.10	−0.26*	0.11	−0.25*	0.11

*N* = 86.

*p < 0.05; **p < 0.01; ***p < 0.001.

*GCI, Global creativity index; GII, Global innovation index.*

**FIGURE 2:**
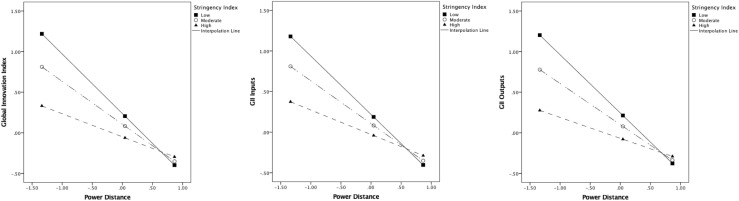
Interaction effect between levels of stringency and Power Distance on innovation indices.

**FIGURE 3:**
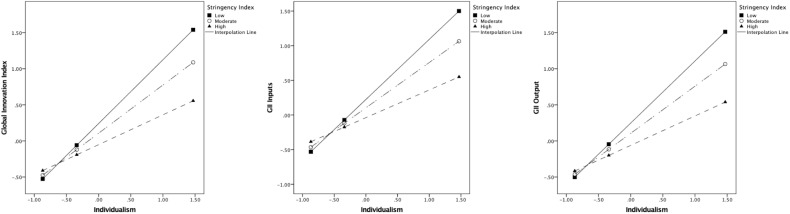
Interaction effect between levels of stringency and Individualism on innovation indices.

Lower power distance and higher individualism predicted greater innovation; moreover, stringency was a salient moderator in the relationship between culture and innovation. The interaction plot (Figure 2) indicated that innovation in countries high on power distance was not associated with stringency levels. However, as stringency increased in countries low on power distance, it was associated with lower general innovation as well as outputs. Similarly, Figure 3 displays the interaction plot between individualism and stringency with the innovation indices. Here, countries with low levels of individualism were associated with lower innovation regardless of stringency. Yet, in highly individualistic countries, greater stringency was related to lower overall innovation, inputs, and outputs.

The same moderation analyses were also run with country-level controls from the World Bank Data and World Development Indicators (age dependency ratio, sex ratio, proportion of urban population, literacy rate, adjusted net national income, and 7-day rolling average of new cases per million per day). However, results indicated that adding the controls crowded out the moderating effect of stringency on the relationship between cultural dimensions and innovation (see Supplementary Tables S1–S3). In these models, literacy, national income, and a greater proportion of males contributed to overall innovation scores. Thus, the moderating effect of stringency was not statistically robust as other predictors of innovation took precedence in explaining variations in GII in the full models.

## Discussion

The onslaught of the COVID-19 pandemic is continuing across the world, unabated. Governments are grappling with how to handle the public health crisis and many countries have imposed strict restrictions on the mobility of their citizens to contain its spread. The outbreak has also severely halted economic activities in nearly all affected countries (Frey et al., 2020). Moreover, this applies to creative and cultural industries, with marked slowdowns in the creative economy (e.g., Comunian and England, 2020). As the pandemic touches new highs every week globally, distress levels rise, prompting individuals, communities, and nations to embark on creative endeavors to make meaning of the ongoing crisis (Kapoor and Kaufman, 2020). Further, there is a large potential for healthcare researchers and policymakers to be creative and innovative in overcoming the pandemic (Cohen and Cromwell, 2020). Against this background, the purpose of this study was to examine how different levels of stringency of government response to COVID-19 interact with certain cultural dimensions in impacting innovation at the country level. Specifically, the findings indicated that stringency moderated the inverse association between power distance and innovation indices, and the direct relationship between individualism and these indices. The dimension of uncertainty avoidance was not related to global innovation, contrary to the first hypothesis.

### Power Distance

Past research has associated high power distance with lower levels of national innovation (e.g., Shane, 1993; Efrat, 2014). Individuals in such countries accept and expect that power will be distributed unequally and often follow a hierarchical structure. For instance, Kirkman et al. (2009) found power distance orientation to consistently predict citizens’ perceptions of a leader’s fair decision-making in both low (United States) and high (China) power distance countries. Extrapolating this finding, it is likely that government actions are not independent of citizens’ expectations. Further, high power distance could impede original thinking and ideation at the individual and national level. In a similar vein, results from this investigation found that high power distance countries had lower innovation, regardless of the extent of government-imposed stringency. It is possible that citizens in such countries hold high expectations from their governments, expecting them to respond as authority figures in handling the situation.

On the other hand, if a country were low on the power distance dimension, but had higher levels of stringency, it was associated with lower national innovation. It is plausible that such nations were unaccustomed to their governments taking strict measures to curb movement and enforcing stay-at-home orders, thereby negatively impacting productive innovative behaviors. It is also possible that citizens in such countries perceive greater stringency by their government as an uncertain aspect of dealing with crisis (Baker et al., 2020). This reduction in innovation was not observed in other low power distance countries with lenient measures to combat the pandemic. When government measures are less severe, citizens may be less burdened with adapting to new changes or routines, thereby enabling them to continue partaking in innovation and creative actions. Overall, the results indicated adverse consequences for global innovation, particularly in countries that had stricter government responses in low power distance countries.

### Individualism-Collectivism

Similarly, high individualism has been associated with higher levels of national innovation (e.g., Khan and Cox, 2017), implying that higher collectivism is related to lower levels of the same (but see Sarooghi et al., 2015). More collectivist cultures represent interdependent social structures, preferring a sense of community over individual needs, and putting others before themselves. Corresponding patterns in the interaction with stringency levels were observed for this cultural dimension as well – irrespective of the levels of stringency, collectivist countries had lower innovation. Research has suggested that social behavior (contingent on culture) may serve as an antipathogenic defense system. These behaviors (like, food preferences) are therefore likely to have proliferated in populations wherein pathogen prevalence has been high. Culturally, it has been observed that collectivism, as opposed to individualism, serves this antipathogenic defense, especially in cultures with higher prevalence of pathogens. This may be associated with the sharp out- and in-group distinction and tolerance for deviance for these two cultural variants wherein outgroup members are seen as potential carriers of pathogens (Fincher et al., 2008). Further research revealed that conformity found in collectivistic cultures partially mediates the relationship between disease prevalence and innovation. Specifically, it was found that although traditionalist and conformist dispositions, found in collectivistic cultures, mitigate the threat of infectious disease, they also dent the opportunities for innovation (Murray, 2014).

Collectivistic cultures predominantly focus on protecting interests of their in-groups, and may strongly disapprove deviation from rules and laws that are meant to protect against the pandemic (Hofstede, 2020). Further, in the middle of a pandemic, it may be perceived to be risky to deviate from societal norms to express originality in such cultures. In contrast, individualistic countries in the sample were more likely to be innovative when stringency levels were low. Greater severity by governments can be interpreted as threatening personal freedoms (Hong et al., 2020) and giving rise to resentment and anger in such countries (Gan et al., 2020), having negative impacts on innovative behaviors. Similar to low power distance countries, individualistic ones with higher stringency may be unfamiliar with government-mandated regulations that impair the *status quo* to such a large extent. Even though a standard lockdown policy may be in place across countries, their cultural make-up can determine individual and collective responses to the current situation. An interplay of lockdown stringency and Hofstede’s cultural dimensions may have an effect on overall innovation in this scenario. Collectivist countries high on power distance may typically find it difficult to innovate due to a threat to the social hierarchy wherein power redistribution can occur. Conversely, individualist countries low in power distance where imagination is rewarded, are likely to be more innovative, but only when government-imposed stringency is low.

The cultural dimension of uncertainty avoidance has been inconsistently linked with creative and innovative output (Shane, 1993; Rinne et al., 2013; Efrat, 2014; Sarooghi et al., 2015). Westwood and Low (2003) point out that although risk-taking has a bearing on creative processes cross-culturally, it does not necessarily translate to the concept of uncertainty avoidance. Yet, individual-level research has identified consistent linkages between ambiguity tolerance and creativity in varied contexts (Giarratana and Torres, 2007; Zenasni et al., 2008; Zhang and Zhou, 2014; Afsar and Masood, 2017). However, creativity and innovation are often expressed (and measured) differently at an individual and national level. Country-level studies measured these constructs in terms of collective output, whereas individual-level studies are at the liberty to measure them with diverse tools (such as self-report instruments and performance-based tasks). The difference between these may contribute to the variance in results for individual and group level data for uncertainty avoidance and originality. Moreover, uncertainty avoidance indicates a desire for personal control wherein one’s life outcomes can be predicted successfully. Therefore, this desire can both help and hinder national innovation, resulting in a zero net effect (Rinne et al., 2013).

### The Need for Global Innovation

Now, more than ever, the world needs innovative and creative solutions to mushrooming problems associated with the pandemic (Azoulay and Jones, 2020). These range from issues in healthcare delivery (Harris et al., 2020) to food distribution (Hawkes, 2020), to the survival of creative and cultural industries (Putrivi, 2020), as well as the survival of pre-existing services and businesses. Innovation has played a salient role in the pandemic until now and will continue to do so until a vaccine has been invented as well as distributed to a sizable portion of the population (Woolliscroft, 2020). Efficient and scalable vaccine distribution is another emerging problem that would require an innovative approach. Similarly, global economic slowdown has been a chief outcome of the COVID-19 pandemic; innovative solutions are needed to jumpstart economies (Greenaway-Mcgrevy et al., 2020) in the face of future projections of even further economic damage (e.g., Demirguc-Kunt et al., 2020).

It may also be important to create online spaces to foster creative and innovative expression to build resilient businesses, communities, and individuals to cope with the pandemic (see also Kapoor and Kaufman, 2020). The problem space is becoming increasingly unique, with civil society organizations, businesses, and artists having to respond with original solutions to keep their audiences engaged. Therefore, it is necessary to identify and acknowledge new problem spaces and areas that demand innovative solutions, as a response to the ongoing pandemic. For instance, one cultural and creative industry that has been directly impacted is music; a report by Seman (2020) outlines the employment and revenue losses incurred by artists in Denver as a result of COVID-19. It also highlights the potential ripples caused in other associated industries, calling on policymakers to respond in a timely manner to ensure the survival of creative activities.

Although this paper does not argue against the importance of stringent government responses to contain the pandemic, it showcases the need to consider such responses in light of a nation’s cultural ethos and background. Based on the present analysis, stringency can deter innovative solutions, which are key in tackling COVID-19.

### Limitations and Conclusion

This study was not without its limitations. First, the inclusion of control variables led to the crowding out of significant relationships, indicating that the results obtained were not robust. However, future studies can use the sub-indices of the GII inputs and outputs to yield more granular analysis; for instance, understanding the impact of stringency on specific components like market sophistication or creative outputs can facilitate the design of intervention strategies to mitigate against a downturn in innovation. Second, data from Hofstede’s dimensions were 2015 estimates; acquiring more recent data on these metrics can result in updated associations between the variables. Third, the stringency index was computed up to September 1, 2020; this was done to maintain consistency with the GII data, which was released in August 2020. Given that the global pandemic and the response to it have been constantly evolving, subsequent research can extend this period of analysis. Fourth, this study examined country-level associations and cannot be used to make inferences at the individual level. To reiterate, creativity and innovation are typically assessed from the individual to the country level and the present investigation can only speak to national-level innovation. Future research can implement instruments that assess adherence to stringency measures, cultural dimensions, and creativity/innovation to explore these relationships for cross-cultural samples. Subsequent research can also compare global creativity and innovation indices in the years before and after the outbreak of COVID-19 to explore temporal changes in these outcomes.

That said, this investigation provided preliminary evidence for the moderating effects of stringency on the inverse relationship between power distance and innovation, and the direct one between individualism and innovation. It may be useful for nations to be cognizant of the impacts of their policies on modern indicators of human development, such as creativity and innovation (e.g., Nobre, 2016), particularly in their response to the public health crisis. When cultures that are not accustomed to adhering to higher authorities, for instance, find themselves in a strict environment, their innovative activity suffers, among other outcomes. By acknowledging the effect of culture on the ease or difficulty of harnessing global innovation and creativity, nations can adjust the strictness of their COVID-19 response.

## Data Availability Statement

Publicly available datasets were analyzed in this study. Data on the GII can be found in the appendices of the 2020 report: https://www.wipo.int/edocs/pubdocs/en/wipo_pub_gii_2020.pdf. Data on cultural dimensions can be found at the Hofstede Insights website: https://geerthofstede.com/researchand-vsm/dimension-data-matrix/. Data on the stringency index can be found at the Oxford Coronavirus Government Response Tracker github page: https://github.com/OxCGRT/covid-policy-tracker/raw/master/data/OxCGRT_latest.csv.

## Author Contributions

HK: conceptualization, methodology, software, formal analysis, writing – original draft, review, editing, and supervision. ATi: conceptualization, resources, writing – original draft, review, and editing. ATa: data curation, formal analysis, software, and writing – review and editing. SK: resources and writing – original draft. All authors contributed to the article and approved the submitted version.

## Conflict of Interest

The authors declare that the research was conducted in the absence of any commercial or financial relationships that could be construed as a potential conflict of interest.

## Footnotes

1 Such cognitive processes comprise original ideation and other relevant mental operations (e.g., Wallas, 1926; Mumford et al., 1991). These creative processes are a pre-requisite for innovative behavior, which is conceptualized within socioeconomic frameworks as the execution of creative ideas (see also Amabile et al., 1996). Throughout the article, research on creativity is reported at the individual psychological level, unless otherwise specified, whereas research on innovation is reported at the group or national level, unless otherwise specified.

2 The most updated data on the Global Creativity index (GCI) were available for 2015 (Florida et al., 2015). Therefore, GCI data were not used in the current analyses. However, theoretically we assume that stringency would moderate the relationship between cultural dimensions and creativity as well.

3 The measurable aspects of the creative economy are typically captured within innovative outputs; for example, creative goods and services such as movies and music (Dutta et al., 2020).

4 It is important to note that over 70% of the data used in the GII innovation measures comes from 2018 to 2019, which precedes the onset of the COVID-19 pandemic.
