# Supplementary material for: Innovation in Isolation? COVID-19 Lockdown Stringency and Culture-Innovation Relationships
Source: Front Psychol. 2021 Feb 3;12:593359. doi: 10.3389/fpsyg.2021.593359 (PMC7886784; doi:10.3389/fpsyg.2021.593359)
Supplement: Supplementary file 1 [file Table_1.pdf]

Supplementary material for Kapoor et al. (2021). Innovation in Isolation? COVID-19 Lockdown Stringency and Culture-Innovation Relationships

Table S1

Regression Analysis Predicting the GII Index from Country-Level Controls and Cultural Dimensions, as Moderated by the Stringency Index

	GII Index					
	B	*SE*	*B*	*SE*	*B*	*SE*
Age dependency ratio	-0.00549	0.104	-0.0198	0.102	-0.0292	0.098
Sex ratio at birth	0.286***	0.064	0.299***	0.060	0.291***	0.059
Urban population as share of total	-0.0205	0.088	-0.0700	0.086	-0.0408	0.083
Adult literacy rate	0.330**	0.098	0.329**	0.097	0.295**	0.098
Adjusted Net national income	0.471***	0.109	0.498***	0.106	0.442***	0.106
Seven-day moving average of new cases per million per day	-0.104	0.063	-0.101	0.059	-0.0934	0.059
Uncertainty Avoidance	-0.0365	0.063				
Stringency Index	-0.0933	0.076				
Uncertainty Avoidance*Stringency	-0.0184	0.071				
Power Distance			-0.0785	0.089		
Stringency Index			-0.0524	0.081		
Power Distance*Stringency			-0.0910	0.104		
Individualism					0.121	0.082
Stringency Index					-0.0494	0.089
Individualism*Stringency					0.0684	0.101
N	56		56		56	
R^2	0.751		0.766		0.769	

Note: Coefficients are from ordinary least square estimates of the GII Index on cultural dimensions, stringency, and control variables. Standard errors in parentheses. *** p<0.001, ** p<0.01, * p<0.05.

Table S2

Regression Analysis Predicting the GII Inputs Index from Country-Level Controls and Cultural Dimensions, as Moderated by the Stringency Index

	**GII - Inputs**					
	B	*SE*	*B*	*SE*	*B*	*SE*
Age dependency ratio	-0.0770	0.111	-0.0704	0.108	-0.0930	0.108
Sex ratio at birth	0.216**	0.069	0.236***	0.064	0.222**	0.065
Urban population as share of total	0.0514	0.095	0.00211	0.091	0.0358	0.091
Adult literacy rate	0.259*	0.106	0.276*	0.104	0.237*	0.108
Adjusted Net national income	0.481***	0.117	0.515***	0.113	0.460***	0.116
Seven-day moving average of new cases per million per day	-0.0980	0.067	-0.0893	0.063	-0.0908	0.065
Uncertainty Avoidance	-0.0274	0.068				
Stringency Index	-0.0977	0.082				
Uncertainty Avoidance*Stringency	-0.0160	0.076				
Power Distance			-0.0287	0.095		
Stringency Index			-0.0372	0.086		
Power Distance*Stringency			-0.158	0.110		
Individualism					0.0815	0.090
Stringency Index					-0.0551	0.098
Individualism*Stringency					0.0705	0.110
N	56		56		56	
R^2	0.738		0.757		0.749	

Note: Coefficients are from ordinary least square estimates of the GII Index on cultural dimensions, stringency, and control variables. Standard errors in parentheses. *** p<0.001, ** p<0.01, * p<0.05.

Supplementary material for Kapoor et al. (2021). Innovation in Isolation? COVID-19 Lockdown Stringency and Culture-Innovation Relationships

Table S3

Regression Analysis Predicting the GII Outputs Index from Country-Level Controls and Cultural Dimensions, as Moderated by the Stringency Index

	**GII - Outputs**					
	B	*SE*	*B*	*SE*	*B*	*SE*
Age dependency ratio	0.0617	0.110	0.0284	0.109	0.0317	0.104
Sex ratio at birth	0.339***	0.069	0.345***	0.065	0.344***	0.062
Urban population as share of total	-0.0873	0.094	-0.135	0.092	-0.111	0.088
Adult literacy rate	0.383***	0.105	0.364**	0.105	0.336**	0.104
Adjusted Net national income	0.441***	0.116	0.461***	0.114	0.407***	0.111
Seven-day moving average of new cases per million per day	-0.105	0.067	-0.107	0.063	-0.0919	0.062
Uncertainty Avoidance	-0.0434	0.067				
Stringency Index	-0.0851	0.081				
Uncertainty Avoidance*Stringency	-0.0198	0.075				
Power Distance			-0.122	0.096		
Stringency Index			-0.0644	0.086		
Power Distance*Stringency			-0.0244	0.111		
Individualism					0.152	0.086
Stringency Index					-0.0420	0.094
Individualism*Stringency					0.0633	0.106
N	56		56		56	
R^2	0.709		0.722		0.736	

Note: Coefficients are from ordinary least square estimates of the GII Index on cultural dimensions, stringency, and control variables. Standard errors in parentheses. *** p<0.001, ** p<0.01, * p<0.05.
